# Epidemiological Trends, Clinical Impact, and Geographical Variations of Nonmelanoma Skin Cancers: A Twelve-Year Study in Messina, Italy

**DOI:** 10.1155/jskc/1823281

**Published:** 2025-06-27

**Authors:** Gabriele Delia, Fabiana Battaglia, Pasqualina Laganà, Giovanni Genovese, Cristina Genovese, Giuseppe Trimarchi, Roberta Giuffrida, Francesco Stagno d'Alcontres

**Affiliations:** ^1^Department of Plastic and Reconstructive Surgery, University Hospital of Messina “AOU Gaetano Martino”, Messina, Italy; ^2^Department of Biomedical, Dental Sciences, and Morphofunctional Imaging, University of Messina, Messina, Italy; ^3^SIR, University of Messina, Messina, Italy; ^4^Department of Clinical and Experimental Medicine, Section of Dermatology, University of Messina, Messina 98125, Italy

**Keywords:** clinical management, geographic variation in cancer, histopathological analysis, nonmelanoma skin cancer, public health intervention, skin cancer epidemiology

## Abstract

The incidence of nonmelanoma skin cancers (NMSCs), including basal cell carcinoma (BCC) and cutaneous squamous cell carcinoma (cSCC), is increasing globally, driven by factors such as chronic UV exposure, climate change, and aging populations. This comprehensive retrospective cohort study examined 1252 patients treated for NMSCs at the Plastic Surgery Unit of the University of Messina, Italy, from 2012 to 2023. The study aimed to assess demographic characteristics, histological types, lesion attributes, recurrence rates, and geographical variations in NMSC incidence. Beyond confirming global epidemiological trends, this study highlights clinically relevant factors influencing tumor presentation, including environmental risk exposure, lesion recurrence, and histological aggressiveness. Data were collected on patient demographics, tumor location, histological subtype, lesion diameter, presence of ulceration, recurrence, and differentiation grade. Statistical analysis was conducted using R software (Version 4.2.0). Findings demonstrated that patients residing in coastal areas had a significantly higher incidence of NMSCs, reinforcing the role of exposure and environmental pollutants in carcinogenesis. These findings align with global trends and highlight the urgent need for early diagnosis, targeted dermatological surveillance, and public health interventions to mitigate the rising burden of NMSCs. The study underscores the necessity of enhanced sun protection awareness, integration of dermatological screenings in primary care settings, and improved treatment protocols to reduce recurrence and morbidity.

## 1. Introduction

Nonmelanoma skin cancer (NMSC) is the most common type of cancer among Caucasian people, with approximately 2.75 million new cases of NMSC detected worldwide each year [1].

The clinical burden of NMSCs continues to escalate, placing significant pressure on healthcare systems. Factors such as chronic sun exposure, climate change, and societal behaviors contribute to the increasing incidence of skin cancers [2]. Skin cancers are classified into two main types: cutaneous melanoma (CM) and NMSCs, which primarily include basal cell carcinoma (BCC) and squamous cell carcinoma (SCC), both of which demonstrate a broad spectrum of clinical presentations and potential complications. In addition, there are several less common conditions such as actinic keratoses (AKs) and keratoacanthoma. Despite being considered treatable, the rising prevalence of NMSCs has made them a significant global health issue [3].

NMSCs, which encompass SCC and BCC, account for up to 25% and 75%–80% of NMSCs, respectively [4, 5]. These cancers are the most frequent neoplasms among the Caucasian population. In the United States, skin cancers were more common than all other neoplasms combined. The incidence of NMSCs is increasing annually, with an estimated 1 in 5 Americans developing skin cancer during their lifetime, 95% of which are NMSCs [4].

Skin tumors were categorized based on the cells from which they originate: melanomas arise from melanocytes, while NMSCs originate from the neoplastic transformation of keratinocytes. BCCs typically form in sun-damaged skin from cells in the lower layers of the skin, while SCCs arise when squamous cells in the epidermis undergo malignant transformation [6].

Cutaneous SCC (cSCC) is involved in the malignant proliferation of keratinizing epidermal cells. It usually develops from precursor lesions such as AKs and Bowen's disease (cSCC in situ) or from skin that has been treated with radiation. Chronically inflamed skin, due to chronic wounds or inflammatory disorders, is also prone to cSCC development.

While BCCs typically exhibit slow progression and lower metastatic potential, cSCCs pose a substantial risk of nodal involvement and systemic spread.

Over the past 30 years, the incidence of cSCC has increased by 50%–200% [7, 8]. Unlike BCC, which rarely metastasizes, cSCC could metastasize to locoregional lymph nodes and distant sites, with metastasis rates estimated between 2% and 5%. This underscores the necessity of aggressive early detection strategies and improved treatment pathways.

Distant metastases are rare and are associated with a poor prognosis and a median survival of less than 2 years, underscoring the importance of early diagnosis and treatment [9].

BCC accounts for 80% of all NMSCs. BCC lesions grow slowly and rarely metastasize but can cause significant morbidity. They often appear on the face, tend to recur, present with multiple lesions, and can invade and damage surrounding tissues. While BCC is generally considered less aggressive, with a lower metastatic potential compared to SCC, it is often the most prevalent form of NMSC.

Epidemiologic studies from various countries have shown a positive correlation between the incidence of NMSC and ultraviolet (UV) radiation exposure and an inverse relationship with skin pigmentation [5]. In addition, recent research highlights the potential influence of environmental pollutants, particularly in coastal regions, on skin carcinogenesis.

### 1.1. cSCC

The exact incidence of cSCC is unknown, and statistics often do not distinguish between the incidence of cSCCs and mucosal SCCs. In Australia, where the highest incidence of NMSCs has been recorded, the overall incidence of cSCC in 2002 was estimated to be 387 cases per 100,000 inhabitants [4]. A recent systematic review of 19 studies examined the trend in the incidence of cSCC in the European White population, demonstrating a marked geographic variation. The highest incidence rates were reported in South Wales (31.7 per 100,000 people per year) and Switzerland (28.9 per 100,000 people per year), while the lowest was in Croatia (8.9 per 100,000 people per year) [10]. cSCC is a rare tumor under the age of 45 years, although the incidence appears to be significantly increasing in younger individuals [11].

In the central and southern United States, deaths from cSCC are as common as those related to oropharyngeal, renal, and melanoma tumors [12].

### 1.2. BCC

Nowadays, the incidence of BCC is difficult to estimate because it is usually not reported in national cancer registries; however, available data indicate geographic variations and that the incidence worldwide continues to increase. In particular, in the Haut region of France, the incidence was estimated to be 75.4 per 100,000 male inhabitants and 60.5 per 100,000 female ones [13]. In the United States, the annual estimated rates are 407 cases per 100,000 male inhabitants and 212 cases per 100,000 female ones [14]. A recent study in Denmark reported an increase in the incidence of BCC from 27.1 to 96.6 cases per 100,000 female inhabitants and from 34.2 to 91.2 cases per 100,000 male inhabitants from 1978 to 2007 [15]. In the Netherlands, the incidence has tripled from 40 to 148 cases per 100,000 male inhabitants and from 34 to 141 cases per 100,000 female inhabitants from 1973 to 2008 [16].

Epidemiological data from Europe showed that NMSCs have an annual incidence rate of 129.3 cases per 100,000 men and 90.8 per 100,000 women [8]. In Italy, the annual incidence of BCC is around 100 cases per 100,000 people, accounting for 15% of all diagnosed malignancies [2]. Messina, located in the northeastern part of Sicily, may be particularly vulnerable to these types of tumors due to its population's high sun exposure, as well as the large number of workers in particular settings with a high level of exposure (summer tourism, agriculture, fisheries, and construction). To assess whether our clinical experience aligns with the broader literature, we conducted a retrospective epidemiological study.

Based on this epidemiological background, this study attempted to assess the incidence of NMSCs and accompanying mortality in Messina. The aim is to evaluate the incidence of NMSCs in Messina by analyzing comprehensive data collected over 12 years, focusing on residents who may be exposed to UV radiation for most of the year.

Based on this epidemiological background, this study aimed to evaluate the incidence and mortality of NMSCs in the Messina area by analyzing comprehensive data collected over a 12-year period, with particular attention to residents chronically exposed to UV radiation. In addition to assessing incidence trends, the study also explored several specific hypotheses: whether the incidence and clinical aggressiveness of NMSCs differ by geographic location (coastal vs. mountainous); whether patient demographics (age and gender) are associated with specific histological subtypes; whether clinical features such as ulceration, lesion size, and recurrence are correlated with tumor type; and whether environmental exposure may influence the development and progression of these lesions. These hypotheses served as the basis for the subsequent analyses presented in this study.

## 2. Materials and Methods

A retrospective cohort study was conducted on 1252 patients treated for NMSC at the Plastic Surgery Unit of the University of Messina, Italy, between 2012 and 2023. This represents an average of approximately 180 patients examined per year.

A total of 2.105 histopathological examinations of NMSCs were retrieved from the Department of Pathology of the AOU “G. Martino.” Demographic data, including age at diagnosis (divided into 10-year age groups), gender, provenance, anatomical district (head and neck, upper limbs, trunk, lower limbs, and genitals) and exact anatomical site, histological type, presence or absence of ulceration, lesion diameter, number of lesions (single or multiple), and presence of recurrences in the same patient were collected for each histopathological examination.

Given the increasing global focus on precision medicine, additional analyses explored correlations between environmental exposure and tumor aggressiveness. Patients were stratified by residence in coastal versus mountainous regions to assess geographic disparities in incidence and severity.

### 2.1. Statistical Analysis

All statistical analyses were performed using the software R (Version 4.2.0) and the package RVAideMemoire.

The non-normality of the age at diagnosis parameter was assessed using the Shapiro–Wilk test, and the summary was expressed as median and IQR. Comparisons with categorical variables (gender, sea/mountain, and ulceration) were made using the Mann–Whitney U test, while the Kruskal–Wallis test was used to compare parameters with multiple categories (CA, dimensions, body area, and histological type). Conover's post hoc test was used when necessary.

Categorical variables (gender, sea/mountain, CA, ulceration, dimensions, histological type, etc.) were presented as absolute frequency and percentage, and associations were made using the chi-square test. An appropriate post hoc test was employed in the presence of multiple tables.

For the purposes of statistical significance, *p* values < 0.05 were considered.

## 3. Results

Among the 1252 patients studied, the majority were male (68.5%), with a mean age at diagnosis of 77 years. A significantly higher proportion of patients resided in coastal areas (70.3%) compared to mountainous regions (29.7%), suggesting a potential association between UV exposure and NMSC incidence (see Table 1).

This geographic difference may reflect increased and prolonged exposure to UV radiation in coastal populations, as well as potential environmental factors such as pollution, which may act synergistically in promoting carcinogenesis. Notably, patients living in coastal areas presented with a higher proportion of larger lesions (> 2 cm), particularly in BCC and cSCC subtypes, further supporting the association between environmental conditions and tumor aggressiveness.

**Figure 1:**
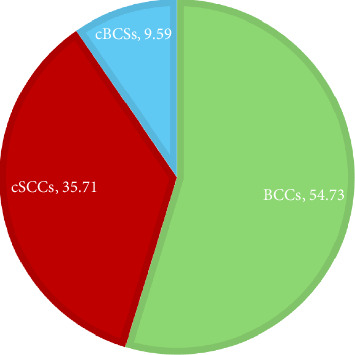
Histological type distribution.

Histological subtypes were distributed as follows: BCCs accounted for 54.73% of cases, cSCCs for 35.71%, and basosquamous carcinomas (cBSCs) for 9.59% (see Figure 1).

The analysis revealed a BCC to cSCC ratio of 1.53, with cBSCs representing 9.59% of cases. Among BCC cases, 440 (64.2%) were male and 245 (35.8%) were female. The cBSC type showed a higher frequency in males (72.5% vs. 27.5%), as did cSCC (73.8% vs. 26.2%).

The most frequent anatomical sites of occurrence were the head and neck regions (84.3%), followed by the trunk (7.4%), lower limbs (4%), and upper limbs (4.3%).

Only one genital lesion was found in a female. BCC and cBSC histotypes were mainly located on the nose, while other significant areas for BCC included the orbital region and the ear. cSCCs were predominantly found on the scalp, ear, and forehead.

Geographically, 880 (70.3%) patients with tumors resided in coastal areas compared to 372 (29.7%) in mountainous regions. The BCC histotype was more common in coastal areas (70.8% vs. 29.2%), as were cBSC (71.7% vs. 28.3%) and cSCC (69.1% vs. 30.9%).

In terms of histotypes within various tumor lesions, the most frequent BCC histotype was nodular (56.49%), followed by sclerodermiform (16.22%) and superficial (3.04%). For cSCC, the most frequent histotype was bowenoid (8.23%), with other forms such as acantholytic, infundibular, and basaloid making up 16.06%.

Ulceration was observed in 53.6% of lesions, with cSCCs exhibiting the highest rates of ulceration (58.1%). Recurrence was documented in 469 patients (37.5%), highlighting the necessity of long-term dermatological follow-up. This finding confirms the more aggressive clinical behavior typically associated with SCCs, particularly in terms of local tissue damage and the need for closer postoperative surveillance (Table 2).

The differentiation grade, crucial for cSCCs, showed that most lesions had a moderate differentiation grade. Multiple lesions were observed in 271 (21.66%) patients, predominantly in males aged 71–80.

Recurrence was noted in 469 patients' postsurgery, with 25 BCC and 16 cSCC cases. Specifically, 1.28% of cSCCs and 2% of BCCs recurred after surgery. The cBSC histotype recurred in 0.64% of surgical cases (8 cases).

Although overall recurrence rates were relatively low (ranging from 0.64% to 2%), their presence underlines the importance of long-term dermatological follow-up, especially in patients with risk factors such as ulceration, advanced age, or multiple lesions.

Further analysis revealed that NMSC patients in coastal areas presented with larger lesions (> 2 cm in 23.50% of BCCs and 25.66% of cSCCs), suggesting that prolonged UV exposure may contribute to more aggressive tumor growth.

Considering the above results, we searched for statistical differences by the different parameters investigated (such as age and gender).

Statistical analysis highlighted several significant associations: males were diagnosed at a younger age than females (*p* < 0.001); BCCs were more frequent in younger patients and associated with smaller lesion diameters (< 1 cm) compared to cSCCs and cBSCs (*p* value < 0.001). Superficial BCCs were more frequently found on the back rather than sun-exposed areas such as the head or arms (*p* value < 0.01), reinforcing the interplay between anatomical site and histological subtype.

Environmental exposure appeared to influence not only tumor frequency but also clinical severity. In coastal populations, lesions tended to be larger, 2 cm in 23.50% of BCCs and 25.66% of cSCCs, suggesting that factors beyond UV radiation, such as air or water pollutants, may contribute to more aggressive presentations.

These observations were further supported by our statistical findings, which showed that residence in coastal areas correlated with higher lesion size and incidence across all histotypes.

Moreover, the BCCs were more frequently associated with a younger age (77) compared to the other two (79 and 80.5) and this resulted also in a lower diameter, less than 1 cm (*p* value < 0.001). Another result is that people with these types of lesions had more frequent lesions on the back than on the head or arms (*p* value < 0.01), especially on superficial skin.

The ulceration was reported more frequently in SCC (*p* value < 0.001).

Moreover, all three types of cancers were more frequently detected in the neck/head.

Age distribution showed that BCCs were most prevalent in the patients aged 71–80, whereas cSCCs and cBSCs were more frequent in those over 80 years old, reflecting the cumulative effect of UV exposure and possibly occupational risk factors more commonly associated with older male populations.

Finally, we investigated the correlation with environmental pollution to understand its potential impact on the prevalence and severity of certain health conditions, aiming to identify key environmental factors that may contribute to disease development and progression.

We observed that patients residing in coastal areas presented with a higher incidence and larger lesions. While direct environmental measurements were not conducted, this pattern may be influenced by multiple factors, including chronic UV exposure and the potential contribution of environmental pollutants, as suggested by prior studies in similar geographic contexts.

## 4. Discussion

The increasing incidence of NMSCs reflects both global trends and localized environmental risk factors [17].

Our findings confirm previous studies demonstrating that coastal populations are at heightened risk, likely due to increased sun exposure and occupational UV risk in industries such as tourism, fisheries, and agriculture. This study also emphasizes the necessity of investigating environmental pollutants as potential co-factors in skin carcinogenesis.

Similar to the findings in other regions, BCC was the most common histological type, accounting for 54.73% of lesions, followed by cSCC at 35.71%. This aligned with the global research indicating that BCC constitutes the majority of NMSC cases [18]; however, our study revealed a rise in SCC occurrences.

An increase in NMSC incidence has been reported worldwide, which is associated with several factors, including a significant aging of the population, increasing the risk of NMSC, and increased sun exposure. Studies have shown that increased occupational and private UV radiation plays a key role in the development of NMSC [17–21]. In women under 40 years of age, a steady increase in BCC incidence was observed [22].

Other studies have shown that tanning beds are associated with a significantly higher risk of BCC and SCC and that, if exposure to artificial radiation occurs by the age of 25, the incidence is even higher [18].

The gender distribution observed, with a higher prevalence of NMSCs in males (68.5%) compared to females (31.5%), aligns with the broader literature indicating that men have a higher incidence of NMSCs, possibly due to greater cumulative sun exposure and less frequent use of sun protection measures compared to women [23].

Age distribution in our cohort also reflects global patterns, with the highest incidence in individuals over 70 years old. This is indicative of the cumulative nature of UV exposure as a risk factor for NMSC [17].

Our analysis of the anatomical distribution of lesions indicated that the head and neck were the most common sites for both BCC and cSCC. This finding is in line with other studies highlighting that these areas receive the most sun exposure, thereby increasing the risk of NMSCs [17]. Notably, BCCs were predominantly found in the nose and orbital regions, while cSCCs were most frequently located on the scalp and forehead.

Interestingly, the data show a higher incidence of NMSC in coastal areas compared to mountainous regions. This geographical variation may be attributed to differing levels of UV exposure due to lifestyle and environmental factors. This finding underscores the importance of targeted public health interventions focusing on sun protection in high-risk areas [24, 25].

Effective preventive strategies should include educational campaigns to raise awareness about the risks of UV exposure and the importance of protective measures such as sunscreen use, wearing protective clothing, and avoiding the peak sun hours. Moreover, regular dermatological screenings in high-risk populations could facilitate early detection and treatment of NMSCs, potentially reducing the incidence and morbidity associated with these cancers.

Due to the high number of cSCCs reported in our case series, the incidence ratio between BCC and cSCC that we observed is considerably lower than the traditionally reported 4:1 ratio in the literature [14, 26]. This observation is consistent with more recent studies, which report ratios of 1.2:1 [27] and 1.4:1 [28].

Our findings emphasize the critical role of early diagnosis and treatment in improving outcomes for NMSC patients. While surgical excision remains the primary treatment modality, other options such as photodynamic therapy and topical imiquimod have shown promise, particularly for BCC. Preventive measures, including regular dermatological check-ups and public education on sun protection, are essential to reduce the incidence and morbidity associated with NMSCs.

This study contributes valuable epidemiological data on NMSCs in the Messina region, aligning with global trends and reinforcing the need for comprehensive strategies to manage and prevent these common skin cancers. Future research should focus on longitudinal studies to monitor incidence trends, genetic and environmental risk factors, and the effectiveness of emerging treatments in diverse populations.

From a clinical perspective, the higher recurrence rates observed in this cohort suggest that current treatment strategies may require optimization. The presence of ulceration and larger lesion diameters in high-exposure areas reinforces the critical need for early intervention, improved surgical approaches, and adjunctive therapies such as topical immunomodulators or photodynamic therapy.

Preventive measures should focus on high-risk populations, advocating for strict sun protection policies and routine dermatological screenings in primary healthcare settings. In addition, emerging therapies targeting molecular pathways involved in NMSC pathogenesis warrant further investigation.

Despite the study's strengths, limitations include its retrospective design and single-center setting. Future multicenter studies with prospective designs could provide more definitive insights into environmental and genetic risk factors influencing NMSC development.

## 5. Conclusion

This study provides essential epidemiological and clinical insights into the growing burden of NMSCs in the Messina region. Findings highlight the urgent need for a multifaceted approach that integrates early detection, personalized treatment, and strategic public health interventions to mitigate disease progression and recurrence.

The observed geographic disparities underscore the importance of tailored dermatological surveillance programs, particularly in high-risk populations with occupational and environmental exposure to UV radiation. Strengthening preventive strategies, such as enhanced sun protection policies, public awareness campaigns, and routine screenings, could significantly reduce morbidity and healthcare costs associated with NMSCs.

Future research should focus on prospective, multicenter studies exploring genetic susceptibility, environmental influences, and emerging therapeutic options to refine risk stratification and improve clinical outcomes. A deeper understanding of these factors will enable the development of more effective, evidence-based interventions aimed at curbing the rising global incidence of NMSCs.

## Figures and Tables

**Table 1:** Sociodemographic characteristics of the investigated sample.

Gender	Males 68.5% (857)		Females 31.5% (395)
Age	< 70 23.7% (297)	71%–80 36.9% (462)	> 80 39.4% (493)
Residents	Coastal areas 70.3% (880)		Mountain areas 29.7% (372)

**Table 2:** Clinical features by tumor type.

Tumor type	Male (%)	Female (%)	Most common age group	Coastal region (%)	Mountain region (%)	Large lesions in coastal patients (%)	Ulceration (%)	Recurrence (%)	Lesions > 2 cm (%)
BCC	64.2	35.8	71–80	70.8	29.2	23.5	N/A	2.0	23.5
cSCC	73.8	26.2	> 80	69.1	30.9	25.66	58.1	1.28	25.66
cBSC	72.5	27.5	> 80	71.7	28.3	N/A	N/A	0.64	N/A

## Data Availability

The data that support the findings of this study are available from the corresponding author upon reasonable request.
